# Supplementary material for: Multi-ethnic Investigation of Risk and Immune Determinants of COVID-19 Outcomes
Source: Res Sq. 2022 Mar 22:rs.3.rs-1055587. Preprint. [Version 1] doi: 10.21203/rs.3.rs-1055587/v1 (PMC8963691; doi:10.21203/rs.3.rs-1055587/v1)
Supplement: Supplement 6 — Supplemental Table 1: Baseline demographic and clinical characteristics of patients presenting to the emergency department, by hospitalization status. [file c8ef79a0531e19f61ae8f9d3.pdf]

Supplemental Table 1: Baseline demographic and clinical characteristics of patients presenting to the emergency department, by hospitalization status.

	Admitted (N=3,086)	Not Admitted (N=1,911)	P-value
**Age (yrs)**	66 (56 - 77)	50 (36 - 62)	<0.001
**Asian**	144 (4.8%)	86 (4.6%)	0.781
**Hispanic**	892 (29.7%)	495 (26.4%)	0.014
**Non-Hispanic Black**	825 (27.4%)	635 (33.9%)	<0.001
**Non-Hispanic White**	689 (22.9%)	397 (21.2%)	0.168
**Other**	458 (15.2%)	262 (14%)	0.245
**Current smoker**	113 (4.7%)	78 (5.5%)	0.282
**Former smoker**	658 (27.4%)	204 (14.4%)	<0.001
**Never smoker**	1629 (67.9%)	1133 (80.1%)	<0.001
**Hypertension**	1096 (35.5%)	258 (13.5%)	<0.001
**Diabetes**	741 (24%)	149 (7.8%)	<0.001
**Coronary artery disease**	395 (12.8%)	78 (4.1%)	<0.001
**Heart failure**	218 (7.1%)	36 (1.9%)	<0.001
**Atrial fibrillation**	201 (6.5%)	36 (1.9%)	<0.001
**Chronic kidney disease**	368 (11.9%)	59 (3.1%)	<0.001
**COPD/asthma**	265 (8.6%)	86 (4.5%)	<0.001
**Obesity**	250 (8.1%)	91 (4.8%)	<0.001
**Cancer**	205 (6.6%)	59 (3.1%)	<0.001
**Chronic liver disease**	83 (2.7%)	28 (1.5%)	0.004
**Obstructive sleep apnea**	65 (2.1%)	19 (1%)	0.003
**HIV**	56 (1.8%)	21 (1.1%)	0.058
**Temperature (°F)**	98.9 (98.2 - 100.2)	98.6 (97.9 - 99.7)	<0.001
**Heart rate (bpm)**	97 (84 - 110)	92 (81 - 104)	<0.001
**Systolic blood pressure (mmHg)**	129 (115 - 146)	131 (119 - 144)	0.02
**Respiratory rate (bpm)**	20 (18 - 22)	18 (17 - 20)	<0.001
**Oxygen saturation (%)**	95 (91 - 97)	98 (96 - 99)	<0.001
**Oxygen sat. <92%**	775 (25.1%)	96 (5%)	<0.001
