# Supplementary material for: Multi-ethnic Investigation of Risk and Immune Determinants of COVID-19 Outcomes
Source: Res Sq. 2022 Mar 22:rs.3.rs-1055587. Preprint. [Version 1] doi: 10.21203/rs.3.rs-1055587/v1 (PMC8963691; doi:10.21203/rs.3.rs-1055587/v1)
Supplement: Supplement 7 — Supplemental Table 2: Baseline demographic and clinical characteristics of patients not admitted to the hospital, by race/ethnicity. [file b4cc53570222dff430f95874.pdf]

Supplemental Table 2: Baseline demographic and clinical characteristics of patients presenting to the emergency department but not admitted to the hospital, by race/ethnicity.

	NH Black (N=635)	NH White (N=397)	Hispanic (N=495))	Asian (N=86)	Other (N=262)
**Age (yrs)**	50 (37 - 61)	52 (35 - 64)	52 (35 - 63)	47.5 (34.25 - 60)	49 (36 - 61)
**Current smoker**	38 (7.5%)	12 (4.3%)	18 (5.2%)	4 (6.2%)	5 (2.5%)
**Former smoker**	55 (10.9%)	46 (16.4%)	68 (19.7%)	5 (7.8%)	29 (14.6%)
**Never smoker**	412 (81.6%)	223 (79.4%)	259 (75.1%)	55 (85.9%)	164 (82.8%)
**Hypertension**	90 (14.2%)	43 (10.8%)	87 (17.6%)	12 (14%)	24 (9.2%)
**Diabetes**	49 (7.7%)	21 (5.3%)	48 (9.7%)	8 (9.3%)	22 (8.4%)
**Coronary artery disease**	19 (3%)	22 (5.5%)	25 (5.1%)	1 (1.2%)	11 (4.2%)
**Heart failure**	10 (1.6%)	7 (1.8%)	15 (3%)	1 (1.2%)	3 (1.1%)
**Atrial fibrillation**	6 (0.9%)	13 (3.3%)	9 (1.8%)	NA	8 (3.1%)
**Chronic kidney disease**	22 (3.5%)	9 (2.3%)	20 (4%)	NA	8 (3.1%)
**COPD/asthma**	34 (5.4%)	10 (2.5%)	33 (6.7%)	NA	8 (3.1%)
**Obesity**	37 (5.8%)	5 (1.3%)	37 (7.5%)	2 (2.3%)	10 (3.8%)
**Cancer**	11 (1.7%)	13 (3.3%)	25 (5.1%)	1 (1.2%)	9 (3.4%)
**Chronic liver disease**	8 (1.3%)	6 (1.5%)	7 (1.4%)	1 (1.2%)	4 (1.5%)
**Obstructive sleep apnea**	10 (1.6%)	1 (0.3%)	4 (0.8%)	NA	4 (1.5%)
**HIV**	5 (0.8%)	2 (0.5%)	12 (2.4%)	NA	2 (0.8%)
**Temperature (°F)**	98.5 (97.8 - 99.6)	98.25 (97.7 - 99.1)	99 (98.3 - 100.4)	98.6 (97.9 - 99.5)	98.7 (98 - 100)
**Heart rate (bpm)**	92 (82 - 102)	88 (78 - 99)	95 (82.5 - 106)	94 (81 - 105.75)	96 (82.25 - 108)
**Systolic blood pressure (mmHg)**	133 (120.5 - 148)	130 (118 - 141)	130 (119 - 144)	129 (118 - 143.5)	129.5 (117 - 140.75)
**Respiratory rate (bpm)**	18 (17 - 19)	18 (17 - 20)	18 (18 - 20)	18 (16 - 19)	18 (18 - 20)
**Oxygen saturation (%)**	98 (97 - 100)	98 (96 - 99)	97 (96 - 98)	98 (97 - 99)	98 (96 - 99)
**Oxygen sat. <92%**	24 (3.8%)	16 (4%)	32 (6.5%)	6 (7.1%)	16 (6.1%)
