# Supplementary material for: Multi-ethnic Investigation of Risk and Immune Determinants of COVID-19 Outcomes
Source: Res Sq. 2022 Mar 22:rs.3.rs-1055587. Preprint. [Version 1] doi: 10.21203/rs.3.rs-1055587/v1 (PMC8963691; doi:10.21203/rs.3.rs-1055587/v1)
Supplement: Supplement 1 — Supplemental Table 4: Univariable logistic regression using demographic and clinical factors to predict in-hospital mortality, stratified by race/ethnicity. [file d41279579edabf660bc7389d.pdf]

Supplemental Table 4: Univariable logistic regression using demographic and clinical factors to predict in-hospital mortality, stratified by race/ethnicity.

Variable	White OR (95% CI)	Black OR (95% CI)	Hispanic OR (95% CI)	All patients OR (95% CI)
**Age (yrs)**	1.07 (1.05-1.08)	1.05 (1.04-1.07)	1.05 (1.04-1.06)	1.06 (1.05-1.06)
**Age 55-64**	1.66 (0.715-3.85)	2.51 (1.27-4.99)	2.77 (1.65-4.66)	2.38 (1.65-3.43)
**Age 65-74**	3.56 (1.75-7.19)	5 (2.61-9.62)	4.22 (2.58-6.97)	4.22 (3.03-5.94)
**Age >=75**	10.3 (5.33-20)	7.92 (4.16-15.1)	6.05 (3.75-9.69)	8.17 (5.91-11.2)
**Race: Non-Hispanic Black**	NA	NA	NA	0.733 (0.589-0.913)
**Race: Hispanic**	NA	NA	NA	0.606 (0.486-0.755)
**Manhattan facility**	0.378 (0.274-0.523)	0.517 (0.379-0.705)	0.593 (0.431-0.816)	0.477 (0.397-0.571)
**Current or former smoker**	1.01 (0.702-1.46)	0.933 (0.662-1.31)	1.46 (1.03-2.06)	1.11 (0.907-1.36)
**Hypertension**	1.39 (0.998-1.94)	1.1 (0.807-1.5)	1.6 (1.17-2.2)	1.32 (1.1-1.58)
**Diabetes**	1.2 (0.795-1.8)	1.57 (1.13-2.19)	1.51 (1.08-2.1)	1.37 (1.12-1.67)
**Coronary artery disease**	1.6 (1.07-2.4)	1.79 (1.16-2.77)	1.65 (1.05-2.58)	1.73 (1.36-2.22)
**Heart failure**	1.85 (1.08-3.16)	1.39 (0.826-2.33)	1.52 (0.864-2.69)	1.59 (1.17-2.17)
**Atrial fibrillation**	1.86 (1.18-2.95)	1.94 (1.02-3.68)	3.06 (1.62-5.83)	2.31 (1.68-3.18)
**Chronic kidney disease**	2.32 (1.33-4.04)	1.75 (1.19-2.59)	1.45 (0.944-2.23)	1.65 (1.28-2.12)
**COPD/asthma**	1.13 (0.652-1.96)	0.634 (0.352-1.14)	0.925 (0.541-1.58)	0.87 (0.634-1.2)
**Obesity**	0.523 (0.262-1.04)	0.817 (0.47-1.42)	1.32 (0.793-2.2)	0.861 (0.62-1.2)
**Cancer**	1.02 (0.566-1.86)	1.15 (0.656-2.02)	1.95 (1.11-3.43)	1.33 (0.959-1.85)
**Respiratory rate**	1.03 (1.01-1.06)	1.1 (1.07-1.14)	1.1 (1.07-1.13)	1.08 (1.06-1.09)
**Oxygen sat. <92%**	2.09 (1.48-2.96)	2.72 (1.89-3.94)	2.71 (1.96-3.74)	2.43 (2-2.95)
