# Supplementary material for: Multi-ethnic Investigation of Risk and Immune Determinants of COVID-19 Outcomes
Source: Res Sq. 2022 Mar 22:rs.3.rs-1055587. Preprint. [Version 1] doi: 10.21203/rs.3.rs-1055587/v1 (PMC8963691; doi:10.21203/rs.3.rs-1055587/v1)
Supplement: Supplement 2 — Supplemental Table 5: Univariable logistic regression using standardized laboratory values to predict in-hospital mortality, stratified by race/ethnicity. [file 9f078faf991a9002b51c80d0.pdf]

Supplemental Table 5: Univariable logistic regression using standardized laboratory values to predict in-hospital mortality, stratified by race/ethnicity.

Test	White OR (95% CI)	Black OR (95% CI)	Hispanic OR (95% CI)	All patients OR (95% CI)
**WBC**	1.62 (1.17-2.24)	1.53 (1.04-2.25)	1.32 (0.994-1.75)	1.48 (1.22-1.79)
**Albumin**	0.589 (0.408-0.85)	0.587 (0.425-0.81)	0.673 (0.513-0.883)	0.624 (0.522-0.747)
**D-dimer**	1.37 (0.971-1.94)	1.17 (0.854-1.59)	1.79 (1.17-2.74)	1.33 (1.1-1.62)
**Ferritin**	1.36 (0.821-2.24)	1.1 (0.858-1.4)	1.18 (0.916-1.51)	1.15 (0.977-1.35)
**Procalcitonin**	1.01 (0.644-1.59)	1.37 (1.01-1.85)	2.71 (0.965-7.63)	1.31 (1.07-1.61)
**LDH**	1.72 (0.951-3.12)	1.3 (0.97-1.73)	1.45 (1.04-2.01)	1.38 (1.13-1.7)
**CRP**	1.65 (1.18-2.3)	1.31 (0.995-1.72)	1.66 (1.24-2.22)	1.52 (1.28-1.81)
**IL-1B**	0.879 (0.515-1.5)	2.06 (1.09-3.87)	0.411 (0.121-1.4)	1.01 (0.774-1.32)
**IL-6**	2.55 (1.36-4.76)	1.39 (1.03-1.87)	1.48 (1-2.18)	1.57 (1.23-2)
**IL-8**	12.9 (1.72-97.9)	7.69 (1.62-36.2)	2.18 (0.792-6.01)	4.31 (1.76-10.5)
**TNF-alpha**	0.999 (0.741-1.35)	1.25 (0.893-1.75)	0.987 (0.314-3.1)	1.09 (0.918-1.3)
