# Supplementary material for: Multi-ethnic Investigation of Risk and Immune Determinants of COVID-19 Outcomes
Source: Res Sq. 2022 Mar 22:rs.3.rs-1055587. Preprint. [Version 1] doi: 10.21203/rs.3.rs-1055587/v1 (PMC8963691; doi:10.21203/rs.3.rs-1055587/v1)
Supplement: Supplement 3 — Supplemental Table 6: Interaction p-values from multivariable logistic regression including interaction terms between race/ethnicity and demographic, clinical, or laboratory factors. [file 46d243e7381de7ff88594886.pdf]

Supplemental Table 6: Interaction p-values from multivariable logistic regression including interaction terms between race/ethnicity and demographic, clinical, or laboratory factors.

Variable	Black vs. White Interaction p-value	Hispanic vs. White Interaction p-value
Age (yrs)	0.157	0.153
Manhattan facility	0.477	0.176
Hypertension	0.938	0.964
Diabetes	0.0885	0.629
Coronary artery disease	0.597	0.833
Heart failure	0.535	0.853
Atrial fibrillation	0.786	0.347
Chronic kidney disease	0.783	0.206
Obesity	0.1	0.0647
Cancer	0.67	0.266
Oxygen sat. <92%	0.831	0.192
**Laboratory Values**		
Albumin	0.72	0.817
CRP	0.473	0.917
D-dimer	0.319	0.707
Ferritin	0.324	0.441
IL-1B	0.038	0.167
IL-6	0.158	0.19
IL-8	0.752	0.0862
LDH	0.266	0.585
Procalcitonin	0.22	0.0734
TNF-alpha	0.573	0.544
WBC	0.954	0.471
