# Supplementary material for: Multi-ethnic Investigation of Risk and Immune Determinants of COVID-19 Outcomes
Source: Res Sq. 2022 Mar 22:rs.3.rs-1055587. Preprint. [Version 1] doi: 10.21203/rs.3.rs-1055587/v1 (PMC8963691; doi:10.21203/rs.3.rs-1055587/v1)
Supplement: Supplement 4 — Supplemental Table 7: Multivariable logistic regression using standardized laboratory values to predict in-hospital mortality, stratified by race/ethnicity, adjusting for age, sex, and baseline hypoxia. [file bfde225fc6010c217a73e351.pdf]

Supplemental Table 7: Multivariable logistic regression using standardized laboratory values to predict in-hospital mortality, stratified by race/ethnicity, adjusting for age, sex, and baseline hypoxia.

var_label	White OR (95% CI)	Black OR (95% CI)	Hispanic OR (95% CI)	All patients OR (95% CI)
**Albumin**	0.698 (0.47-1.04)	0.681 (0.479-0.968)	0.769 (0.568-1.04)	0.745 (0.612-0.906)
**CRP**	1.613 (1.12-2.33)	1.266 (0.954-1.68)	1.445 (1.04-2.01)	1.392 (1.16-1.68)
**D-dimer**	1.283 (0.878-1.88)	0.911 (0.62-1.34)	1.398 (0.892-2.19)	1.141 (0.917-1.42)
**Ferritin**	1.484 (0.886-2.49)	1.13 (0.866-1.47)	1.204 (0.885-1.64)	1.186 (0.991-1.42)
**IL-1B**	0.782 (0.405-1.51)	2.349 (1.13-4.86)	0.254 (0.0596-1.07)	0.977 (0.741-1.29)
**IL-6**	2.296 (1.21-4.36)	1.302 (0.949-1.79)	1.274 (0.837-1.94)	1.43 (1.12-1.82)
**IL-8**	8.758 (0.953-80.7)	6.05 (1.19-30.3)	1.514 (0.594-3.86)	2.858 (1.14-7.17)
**LDH**	1.808 (0.945-3.46)	1.183 (0.866-1.61)	1.359 (0.95-1.95)	1.344 (1.07-1.68)
**Procalcitonin**	0.981 (0.581-1.66)	1.383 (0.999-1.91)	2.654 (0.884-7.96)	1.27 (1.01-1.59)
**TNF-alpha**	0.974 (0.66-1.44)	1.132 (0.798-1.61)	0.71 (0.172-2.93)	1.052 (0.863-1.28)
**WBC**	1.478 (1.06-2.07)	1.399 (0.898-2.18)	1.172 (0.879-1.56)	1.335 (1.08-1.65)
