# Supplementary material for: Multi-ethnic Investigation of Risk and Immune Determinants of COVID-19 Outcomes
Source: Res Sq. 2022 Mar 22:rs.3.rs-1055587. Preprint. [Version 1] doi: 10.21203/rs.3.rs-1055587/v1 (PMC8963691; doi:10.21203/rs.3.rs-1055587/v1)
Supplement: Supplement 5 — Supplemental Table 8: Median cytokine levels of patients from the University of Pennsylvania cohort. [file f4d767c2ab48a0d9b5b31ef7.pdf]

Supplemental Table 8: Median cytokine levels of patients from the University of Pennsylvania cohort.

Cytokine	Healthy Donor (HD, N=10)	Recovered Donor (RD, N=12)	HD+RD (N=22)	NH Black (NHB, N=8)	Asian (AS, N=4)	NH White (NHW, N=3)	P-value (NHB vs. HD+RD)	P-value (NHW vs. HD+RD)	P-value (AS vs. HD+RD)
IL1beta	2.20	3.88	2.66	4.13	4.44	3.8	0.115	0.138	0.090
IL6	2.25	2.59	2.53	37.1	54.6	211	**0.003**	0.157	0.094
TNFa	14.2	12.6	13.7	9.47	7.37	9.15	0.165	0.086	0.231
IP10	77.2	55.6	61.5	227	266	509	**0.006**	**0.041**	**0.025**
IFNlamda1	82.5	82.1	82.1	71.1	90.7	49.0	0.659	0.863	0.514
IL8	8.25	6.74	7.60	18.5	12.8	36.2	0.659	0.157	0.094
IL12p70	3.14	3.42	3.20	1.98	1.78	4.57	**0.004**	0.707	0.513
IFNa2	2.72	4.82	3.49	5.06	4.92	3.90	0.638	0.707	0.497
IFNlamda2.3	12.2	15.2	13.7	12.3	12.0	12.9	0.693	0.598	0.693
GMCSF	11.7	7.15	8.32	6.57	7.92	8.44	0.296	0.598	0.693
IFNbeta	4.25	4.25	4.25	7.07	5.39	4.25	0.650	0.985	0.260
IL10	12.3	9.34	10.5	15.5	18.8	18.3	0.189	0.117	0.069
IFNg	5.97	7.91	6.04	7.32	8.71	7.36	0.296	0.328	0.231
